# Successful Treatment of Acromegaly and Associated Hypogonadism with First-Line Clomiphene Therapy

**DOI:** 10.1155/2018/7925019

**Published:** 2018-06-26

**Authors:** Juan D. Palacios, Ricardo J. Komotar, Atil Y. Kargi

**Affiliations:** ^1^Diabetes & Endocrinology Institute at Renaissance, Edinburg, TX 78539, USA; ^2^Division of Neurosurgery, University of Miami Miller School of Medicine, Miami, FL 33136, USA; ^3^Division of Endocrinology, Diabetes and Metabolism, Department of Medicine, University of Miami Miller School of Medicine, Miami, FL 33136, USA

## Abstract

Clomiphene citrate (CC) has been reported as an effective add-on therapy to somatostatin analogs and dopamine agonists in patients with acromegaly accompanied by hypogonadism; its use as a single agent to treat acromegaly and associated hypogonadism following incomplete surgery has not been previously reported. We report the first case in which clomiphene was utilized as a single agent for the dual management of acromegaly and hypogonadism, not controlled by pituitary surgery alone. The treatment was well tolerated and proved to be effective after a process of treatment withdrawal and reintroduction. We propose that clomiphene may be considered as a cost-effective oral treatment option in select cases of hypogonadal acromegaly.

## 1. Introduction

Hypogonadism is commonly observed in men with acromegaly, reported in 54% of patients with macroadenomas and 38% patients with microadenomas [1]. The potential mechanisms explaining the occurrence of hypogonadism in acromegaly include mass effect with compression of the pituitary gland or stalk and hyperprolactinemia.

Transsphenoidal surgery (TSR) is the primary therapy in most cases of acromegaly; the remission rate following surgery is 80-90 % for microadenomas and 40–50 % for macroadenomas [2]. Additional options for treatment of acromegaly include somatostatin receptor ligands (SRL), growth hormone receptor antagonists (GHRA), and dopamine agonists and radiation. A recent study reported efficacy of clomiphene citrate (CC) as an add-on therapy for men with acromegaly not controlled by existing therapies and either low or low-normal serum testosterone [3]. However, there are no existing reports of the effects of CC as single agent or first-line therapy for hypogonadism in males with persistent acromegaly accompanied by hypogonadism following TSR.

## 2. Case Presentation

**Figure 1:**
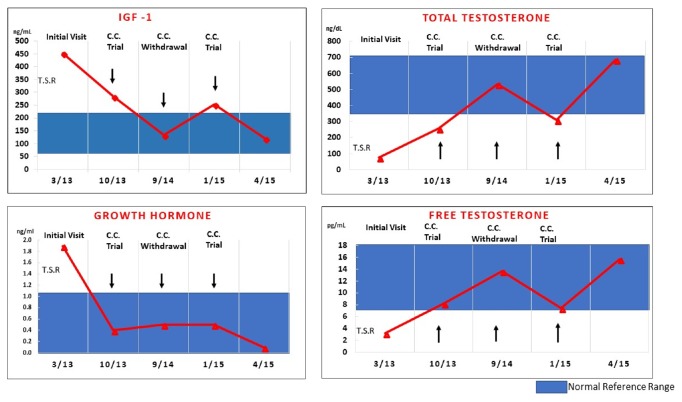
Timeline of treatment effects on serum IGF-I, growth hormone (GH), testosterone, and free testosterone levels. TSR: transsphenoidal resection. CC: clomiphene citrate.

A 49-year-old male was referred for treatment acromegaly and hypogonadism secondary to a suspected GH and prolactin cosecreting pituitary adenoma. He endorsed symptoms of 4-year duration including fatigue, decreased libido, increased snoring, prognathism, increased ring, and shoe size and arthralgias. Physical exam demonstrated a mildly overweight man with normal blood pressure and minimal physical signs of acromegaly who appeared well virilized and exhibited normal testicular size. Serum IGF-I level by immunochemiluminometric assay (ICMA) was increased at 457 ng/mL (reference range 67-205 ng/mL), as well as serum prolactin 79 ng/ml (reference range 4.0–15.2 ng/ml) growth hormone (GH) level 1.9 ng/mL (reference range 0-10 ng/mL) which did not suppress 2 hours after a 75 gram oral glucose load measured at 1.8 ng/mL; while serum testosterone level was markedly decreased at 74 ng/dL (reference range 348-1197ng/dL), gonadotropin levels were normal (Figure 1). MRI demonstrated a 9 x 9 mm mass lesion in the left inferior-posterior aspect of the sella turcica abutting the left carotid artery. The patient was treated initially with transsphenoidal surgery achieving a gross total resection. Histopathology revealed a pituitary adenoma staining strongly on immunohistochemistry for prolactin and weakly for GH.

Six months after surgery, serum IGF-I level was persistently elevated at 285ng/mL; a random GH level was 0.4 ng/mL. Prolactin levels were no longer elevated and the remainder of the pituitary function tests was normal except for serum total testosterone which remained low at 255 ng/dl and low free testosterone measured by mass spectrometry at 6.0 pg/ml (reference range 6.8-21.5.pg/ml). MRI showed a partial empty sella with no evidence of tumor. The patient reported improvement of joint aches, snoring and swelling of hands yet complained of persistent fatigue and sexual dysfunction. After discussion of treatment options including somatostatin analogs, dopamine agonists, growth hormone receptor antagonist, and various forms of testosterone replacement, the patient preferred to attempt a trial of clomiphene citrate. The patient was started on oral clomiphene citrate (CC), 25 mg/day.

Six months after clomiphene initiation, the patient reported resolution of fatigue and improved libido which correlated with an improvement on total testosterone levels 355 ng/dL and free testosterone level 8.6 pg/ml. 11 months after clomiphene therapy, laboratory evaluation reported normalization of both serum IGF-I (134 ng/ml) and a peak serum total testosterone (535ng/dl) and free testosterone (12.6 pg/ml), while GH 0.5 ng/ml and prolactin levels (5.5 ng/ml) remained controlled (Figure 1). Free testosterone levels increased into the normal range with CC treatment, paralleling the increase of total testosterone levels (Figure 1). After a brief trial of four months of discontinuing clomiphene treatment, total testosterone, free testosterone, and IGF-I levels returned to near pretreatment values and the patient had recurrence of hypogonadal symptoms. Upon resuming clomiphene therapy, testosterone and IGF-I levels returned to target, fulfilling “Koch's postulate” providing evidence of effectiveness of therapy.

## 3. Discussion

Clomiphene citrate (CC) is a SERM that possesses positive estrogenic effect on peripheral tissues, while exhibiting “central” estrogen antagonism at the level of the hypothalamus and pituitary, thus increasing LH and FSH secretion and improving hypogonadism and fertility outcomes. Several clinical studies have reported efficacy and safety of CC for the treatment of men with secondary hypogonadism from a variety of causes including prolactinoma [3].

The role of estrogen in reducing IGF-I by antagonizing GH receptors is well known. Estrogen regulates the metabolic effects of GH not just at the level of secretion, but also at the level of GH action by several mechanisms including (1) reduction of the expression of GH receptors on the cells, (2) upregulation of suppressors of cytokine signaling-2 (SOCS2) thereby impairing GH-induced Janus kinase 2 phosphorylation and attenuating intracellular GH signaling thereby reducing IGF-I production, and (3) suppression of GH receptor signaling throughout nongenomic pathways by inducing phospholipase C activation that could cause desensitization of Janus kinase/STAT signaling due to reduced availability of STAT proteins in the cytoplasm [4].

Oral estrogen treatment as either OCPs or postmenopausal HRT has been reported to lower IGF-1 levels in women with acromegaly [5]. Several short-term clinical studies have reported lowering of serum IGF-1 in acromegalic males by treatment with SERMs including tamoxifen and raloxifene. Raloxifene lowered circulating IGF-1 without altering GH levels and did not significantly affect testosterone levels in a 5-week study of primarily nonhypogonadal male acromegalics [6, 7]. Prior to this report, CC has only been reported as an third-line medical agent for hypogonadal acromegalic men with a recent study reporting efficacy of CC as an add-on therapy for men with acromegaly not controlled by existing therapies and either low or low-normal serum testosterone levels resulting in a 44 % reduction in mean IGF-I and 209% increase in mean serum testosterone concentrations [2].

We report the first description of successful treatment with CC as first-line single agent therapy to achieve biochemical and clinical control of both acromegaly and hypogonadism following noncurative pituitary surgery in an acromegalic man. The treatment was well tolerated and proved effective after a careful process of treatment withdrawal and reintroduction. Given the substantial costs of common treatment options for acromegaly and hypogonadism as well as the need for parenteral administration of such treatments, we suggest that clomiphene may be considered as a cost-effective treatment option in selected cases of male hypogonadal acromegaly. While excess GH is known to decrease circulating sex-hormone binding globulin (SHBG) and estrogen or SERMs may increase SHBG, in our patient changes in free testosterone levels paralleled those of total testosterone, ruling out the possibility that the improvement in total testosterone concentration was due mainly to an effect of CC to increase SHBG (Figure 1). Since there is a “first pass” effect of oral CC on the liver, which is the primary source of circulating IGF-I, it is possible that CC treatment may in effect “mask” persistent acromegaly at the level of peripheral tissues where local IGF-I production could remain increased, despite normalization of hepatic-derived serum IGF-I.

CC should be considered primarily for treatment of symptomatic secondary hypogonadism with a secondary benefit to lower IGF-I levels. It should be kept in mind that CC does not suppress GH production and it is unknown to what degree CC antagonizes GH effects other than IGF-I production. Patient preference for cost and mode of therapy may provide additional guidance as CC is an inexpensive, safe oral therapy option for hypogonadal men. We propose the following criteria for the consideration of CC monotherapy in acromegaly associated hypogonadism: (1) minimal or no residual tumor on MRI, (2) GH < 1 ng/ml, (3) mild elevation of IGF-I, and (4) concurrent secondary hypogonadism without panhypopituitarism. Before CC can be recommended as a routine treatment option in hypogonadal acromegaly, long-term studies of clomiphene or other SERMs will be necessary to assess efficacy and safety, outcomes related to “hard” end-points including acromegaly symptom scores and acromegaly related morbidity and mortality, and potential for pituitary tumor growth due to reduction of IGF-I feedback.
